# 

**Published:** 1887-04-30

**Authors:** 


### CONTENTS.

**Death against Doctors** - - - - - 67

**Words of Consolation.**—XXXI. Death - - 68

**Nurse Marion's Romance.** By KATE FURLEY. Chapter VI. The Widow Explains - - - 69

**One Year Probationers** - - - - - 70

**"The Hospital's" Nursery Rhymes** - - 70

**The Hospital Saturday Fund.** By ONE OF THE MASSES - - - - - - - - 71

**The Medical Student's Complaint** - - 72

**A National Pension Fund for Hospital Officials and Nurses.** The Views of Officials, Sisters, Nurses, and Others - - - - - 73

**Notes and News.** — Hospital Appointments. — The Liverpool Foundling Hospital. — Miscellaneous Hospital Intelligence.—Vacancies.— Morley House Seaside Home.—Entertainments in Aid of Guy's Hospital.—Donations, Legacies, etc., to Medical Charities.— The Pembrokeshire and Haverfordwest Infirmary, etc. 74

**Annotations.**—Ambulance and Self-help at Leeds.— Still Unreformed.—Grumblers - - - - - 77

**Till the Doctor Comes.** — XVII. Infection and Disinfection - - - - - - - 78

**The Ventilation of Hospitals.** By Sir ANDREW CLARK, Bart, M.D.; Sir JOSEPH FAYRER. K.C.S.I., M.D.; Dr. SYMES THOMPSON; Dr. THEODORE WILLIAMS; Mr. BURDETT, AND OTHERS - - - 79

**The Artificial and Natural Ventilation of Hospitals.** By KEITH D. YOUNG, F.R.I.B.A. - 82

**Incidents of Animal Life.**—A Noble Dog - - 82

**The Children's Corner.**—Puzzles, Answers, etc. - 83

**Amusements with Profit** - - - - . 83

**In- and Out-Patients' Hospital Diary** - - 84

